# Sleep duration, sarcopenia, and incident depressive symptoms in Chinese middle-aged and older adults: A prospective cohort study based on CHARLS

**DOI:** 10.1097/MD.0000000000051177

**Published:** 2026-10-23

**Authors:** Jiaran Jiang, Quan Zhou, Chuanxia Zhang, Yiming Ma

**Affiliations:** a Department of General Education, Shandong Xiehe University, Jinan, Shandong, China; b Graduate School of Arts and Science, Gachon University, Seongnam, Gyeonggido, Republic of Korea.

**Keywords:** depressive symptoms, mediation analysis, prospective cohort study, Sarcopenia, sleep duration

## Abstract

Sarcopenia and depressive symptoms are prevalent health issues in middle-aged and older adults, yet the mechanisms linking them remain unclear. This study aimed to investigate the association between sarcopenia and incident depressive symptoms and to examine the potential mediating role of sleep duration. We conducted a prospective cohort study using data from the China Health and Retirement Longitudinal Study from 2011 to 2015. A total of 5346 participants aged 45 years and older without baseline depressive symptoms were included. Sarcopenia was defined according to the Asian Working Group for Sarcopenia 2019 criteria. Depressive symptoms were assessed using the 10-item Center for Epidemiological Studies Depression Scale, with a cutoff of ≥10 indicating depression. Sleep duration was self-reported. Cox proportional hazards models, restricted cubic spline regression, and bootstrap mediation analysis were employed. During a 4-year follow-up, 1482 participants (27.72%) developed incident depressive symptoms. After adjusting for confounders, both possible sarcopenia and confirmed sarcopenia were significantly associated with an increased risk of depressive symptoms (hazard ratio [HR] = 1.21, 95% confidence interval [CI]:1.02–1.44 and HR = 1.36, 95% CI:1.04–1.78, respectively). Longer sleep duration was independently associated with a lower risk of depression (HR = 0.93, 95% CI: 0.90–0.96). However, the mediating effect of sleep duration became non-significant after covariate adjustment (*P* = .302). Sarcopenia was independently associated with incident depressive symptoms, but sleep duration did not significantly mediate this association after adjustment. These exploratory findings should be interpreted cautiously. These findings suggest that interventions targeting sarcopenia prevention and management may confer beneficial effects on mental well-being in aging populations.

## 1. Introduction

The accelerating global aging population has heightened the focus on sarcopenia as a major condition strongly associated with poor health in middle-aged and older adults. Sarcopenia is defined as an age-related degenerative musculoskeletal disorder, characterized by a progressive loss of skeletal muscle mass, decreased muscle strength, and reduced physical performance, significantly compromising functional independence and quality of life in older adults.^[1]^ Sarcopenia poses multifaceted health threats to the aging population. Firstly, it markedly increases the risk of losing functional independence and frailty. For instance, impairments in physical capacity, such as reduced grip strength, further elevate the likelihood of falls, disability, and dependence on long-term care.^[2]^ Secondly, sarcopenia is closely associated with a variety of adverse health outcomes. In the cardiovascular domain, it is significantly linked to an increased risk of cardiovascular disease.^[3]^ A study based on the Asian Working Group for Sarcopenia (AWGS) 2019 criteria demonstrated a higher risk of cardiovascular disease incidence (hazard ratio [HR]: 1.22–1.33) among affected individuals.^[4]^ The condition also exhibits a synergistic association with cognitive impairment and dementia, further diminishing the capacity for activities of daily living.^[5]^ Moreover, sarcopenia is significantly associated with elevated all-cause mortality, a predictive value that becomes even more pronounced when comorbid with malnutrition.^[6,7]^

Sleep duration is strongly associated with the health of middle-aged and older adults. Research indicates that short sleep duration (<7 hours) during midlife (ages 50–70) is significantly associated with a 30% increased risk of late-onset dementia, an association independent of sociodemographic, behavioral, cardiometabolic, and mental health confounders.^[8]^ Furthermore, long-term fluctuations in sleep duration also significantly elevate the risk of cognitive impairment.^[9]^ Regarding cognitive function, the combined patterns of nocturnal sleep duration, sleep midpoint, and sleep onset latency exhibit independent and joint associations with global cognitive performance. In terms of mental health, both short (<7 hours) and long (>8–9 hours) sleep duration, as well as insomnia symptoms, are significant risk factors for depression in older adults. A well-established bidirectional relationship exists between depression and sleep abnormalities, where each condition can predispose to and exacerbate the other.^[10]^ Regarding physical health, abnormal sleep duration (either insufficient or excessive) is associated with an increased risk of developing initial chronic diseases and multimorbidity. Specific risk thresholds appear to be age-dependent, highlighting the nuanced role of sleep across the lifespan.^[11]^ These findings underscore the paramount importance of appropriate sleep duration for maintaining physical health.

As one of the most common mood disorders among middle-aged and older adults, depression significantly impairs quality of life and plays a critical role in the relationship between sarcopenia and frailty. Across different sarcopenia status groups, depression consistently associated with frailty. Mediation analyses further indicate that sarcopenia is not only associated with frailty through depression and cognitive function separately, but also associated with frailty through a chain-mediated pathway involving both depression and cognitive function.^[12]^ A significant association also exists between sleep duration and depression.^[13]^ Against this background, the present study proposes that sleep may serve a mediating role in the association between sarcopenia and depressive symptoms in middle-aged and older adults. Therefore, systematically investigating the potential mediating role of sleep in the relationship between sarcopenia and depressive symptoms will not only contribute to a deeper understanding of their underlying connection but may also provide theoretical support for developing comprehensive intervention strategies for the aging population.

## 2. Materials and methods

### 2.1. Data source

The China Health and Retirement Longitudinal Study (CHARLS) is designed to collect high-quality data from a nationally representative sample of Chinese adults aged 45 and older to support research on aging-related issues. The baseline survey was conducted in 2011, with follow-up surveys carried out approximately every two years, forming a longitudinal panel dataset with time-series characteristics. Further details on the study’s methodology are available on the CHARLS project website (http://charls.pku.edu.cn/). Prior to participation, all subjects provided written informed consent, and the Institutional Review Board of Peking University (IRB00001052–11015) approved the study. All procedures involving human subjects adhered to the guidelines of the institutional research committee and followed the principles outlined in the 1964 Declaration of Helsinki. The data accessed for this research were obtained from the CHARLS public database on February 1, 2025. Throughout the data analysis and manuscript preparation, the authors had no access to any information that could identify individual participants.

### 2.2. Population selection

This study utilized data from the CHARLS 2011 baseline survey, and the participant selection process is detailed in Figure 1. Briefly, the survey initially included 17,708 respondents. Among them, a total of 13,565 participants completed all three waves of follow-up (2011, 2013, and 2015). After applying exclusion criteria, 394 participants aged below 45 years were removed. An additional 3667 participants were excluded due to missing sarcopenia screening indicator data (n = 3219) or key covariates (n = 448). A further 4158 participants were excluded based on baseline depression. Following these exclusions, the final analytical sample consisted of 5346 participants. Given the complex missingness patterns in the CHARLS dataset: particularly the fact that sarcopenia components (muscle mass, grip strength, and physical performance) were not missing completely at random: and the need for complete-case analysis to maintain consistency with the AWGS 2019 diagnostic algorithm, we opted for complete-case analysis with inverse probability weighting (IPW) to mitigate selection bias.

**Figure 1.**
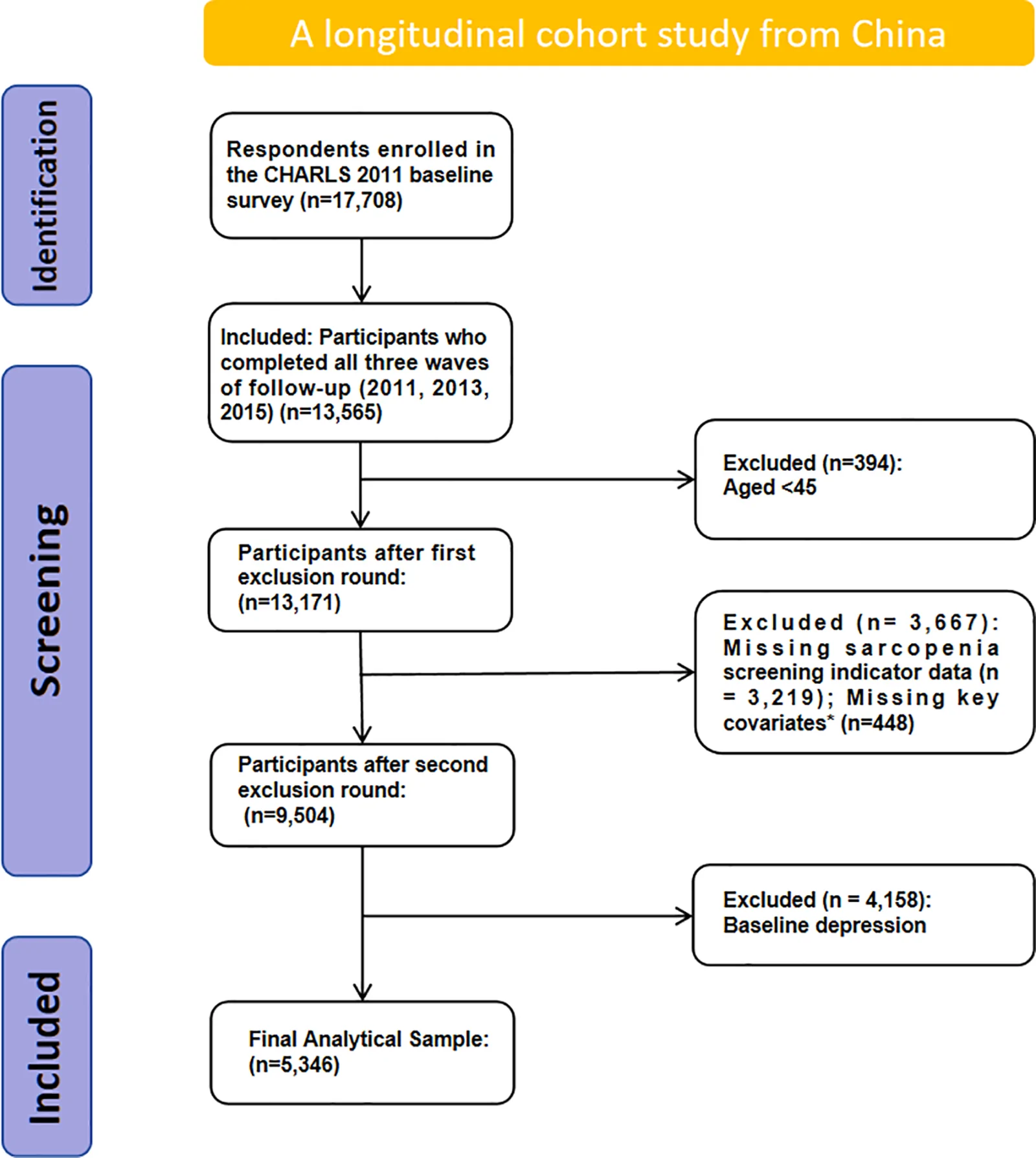
Flowchart of participant selection from the China Health and Retirement Longitudinal Study (CHARLS, 2011 to 2015). (Key covariates*: gender, age, rural, marital status, education, smoking, drinking, chronic diseases, BMI). BMI = Body mass index, CI = confidence interval, HR = hazard ratio.

### 2.3. Measures

#### 2.3.1. Exposure variable selection and assessment

Sarcopenia was diagnosed and assessed for severity according to the 2019 AWGS 2019 criteria.^[14]^ The diagnostic criteria encompass three core dimensions: muscle strength, muscle mass, and physical performance. Muscle strength was evaluated using handgrip strength (HGS), measured with a handheld electronic dynamometer. Participants performed maximum effort trials with their dominant hand, and the cutoff values for low muscle strength were set at <28 kg for men and <18 kg for women. Physical performance was primarily assessed using the Short Physical Performance Battery (SPPB). A score of ≤9 on the SPPB, a 4-meter gait speed <1.0 m/s, or a time ≥12 seconds for the five-repetition sit-to-stand test were used as indicators of reduced physical performance. Muscle mass was estimated using an anthropometry-based equation for appendicular skeletal muscle mass (ASM), which has been validated in the Chinese population and shows high agreement with dual-energy X-ray absorptiometry results.^[15]^ The equation is as follows: ASM = 0.193 × weight (kg) + 0.107 × height (cm) − 4.157 × sex (male = 1, female = 0) − 0.037 × age −2.631. Height and weight were measured using a Seca TM213 stadiometer and an HN-286 scale, respectively. The appendicular skeletal muscle mass index (ASMI), kg/m^2^ was then calculated by adjusting ASM for height squared (ASM/height^2^). Low muscle mass was defined using cohort-specific cutoff values (sex-specific 20th percentile): ASMI ≤ 6.79 kg/m^2^ for men and ASMI ≤ 4.90 kg/m^2^ for women.^[16]^

#### 2.3.2. Covariates of interest

Covariates included age, sex, marital status (married/cohabiting vs unmarried), residence (urban vs rural), presence of chronic diseases (yes/no), smoking status (current vs never), alcohol use (yes/no), and educational attainment (≥high school, junior high school, or ≤primary school/illiterate). Body mass index (BMI) was calculated from measured height and weight and categorized as underweight (<18.5 kg/m^2^), normal weight (18.5–23.9 kg/m^2^), or overweight/obese (≥24 kg/m^2^).

#### 2.3.3. Outcome measurements

Depressive symptoms were assessed as the primary outcome using the 10-item Center for Epidemiological Studies Depression Scale (CESD-10), as implemented in the CHARLS database. Each item is scored from 0 to 3, yielding a total score ranging from 0 to 30, with higher scores indicating more severe depressive symptoms. In line with the CHARLS protocol, a total score ≥10 was used to define the presence of depressive symptoms, while scores <10 were considered normal.

### 2.4. Statistical analysis

All statistical analyses were conducted using R software (version 4.3.0). Continuous variables following a normal distribution are presented as mean ± standard deviation, and comparisons among multiple groups were performed using one-way analysis of variance. Categorical variables are expressed as frequency and percentage, and group comparisons were carried out using the chi-square test. A two-sided *P*-value < .05 was considered statistically significant. Three progressively adjusted Cox proportional hazards models were constructed to assess the associations between sarcopenia, sleep duration, and the risk of depressive symptoms: Model 1: Unadjusted. Model 2: Adjusted for age, sex, educational level, marital status, and residence (urban/rural). Model 3: Further adjusted for smoking, alcohol use, chronic diseases, and BMI. All Cox regression analyses were performed using IPW. The proportional hazards assumption for all Cox regression models was assessed by examining Schoenfeld residuals. No significant violations were detected (all *P* > .05), confirming that the assumption was met for all covariates. Sampling weights provided by CHARLS were incorporated to ensure the national representativeness of the results. Restricted cubic spline (RCS) regression was employed to examine potential nonlinear relationships between indicators of sarcopenia (ASMI, HGS, SPPB) and the risk of depressive symptoms. Subgroup analyses were performed according to predefined demographic, lifestyle, and health-related factors to evaluate effect heterogeneity. To minimize reverse causality, participants with depressive symptoms at baseline were excluded. Variance inflation factors (VIFs) were calculated to assess multicollinearity among covariates; a VIF > 5 was considered indicative of critical collinearity. Mediation analysis was conducted to exploratorily evaluate the potential mediating role of sleep duration in the association between sarcopenia and depressive symptoms. Given the cross-sectional measurement of both exposure and mediator at baseline, this analysis should be considered hypothesis-generating rather than confirmatory. The mediation proportion was calculated, and the significance of the indirect effect was estimated using 1000 bootstrap samples. Path coefficients (a, b, c, c′) were estimated, and 95% confidence intervals (CIs) were computed to assess direct and indirect effects. To assess whether the main findings were sensitive to the dichotomization of depressive symptoms, we repeated the analyses using the continuous CESD-10 score as the outcome in linear regression models.

## 3. Results

### 3.1. Baseline characteristics of the study population

As shown in Table 1, this study ultimately included 5346 participants with a mean age of 57.22 years, among whom 49.57% were female. According to the sarcopenia criteria, participants were categorized into three groups: non-sarcopenia (n = 4563), sarcopenia (n = 160), and possible sarcopenia (n = 623). The majority of participants were married (92.33%), resided in rural areas (64.27%), did not consume alcohol (64.31%), and were nonsmokers (68.35%). Educational attainment was predominantly at the level of primary school or below (40.74%). Regarding BMI categories, 51.66% were of normal weight (18.5–23.9 kg/m^2^), 4.77% were underweight (<18.5 kg/m^2^), and 43.57% were overweight/obese (≥24 kg/m^2^). A total of 1482 participants developed incident depressive symptoms during the study period. Furthermore, the sensitivity analysis revealed no significant multicollinearity (maximum VIF = 1.99 < 5), supporting the robustness of the multivariate estimates (Table A1, Supplemental Digital Content 1).

**Table 1:** Baseline characteristics of participants grouped by sarcopenia status.

Variables	Total (n = 5346)	Non sarcopenia(n = 4563)	Possible sarcopenia(n = 160)	Sarcopenia(n = 623)	*P* value
Gender, n(%)					.359
Male	2696 (50.43)	2305 (50.52)	72 (45.00)	319 (51.20)	
Female	2650 (49.57)	2258 (49.48)	88 (55.00)	304 (48.80)	
Marry, n(%)					<.001
Others	410 (7.67)	279 (6.11)	40 (25.00)	91 (14.61)	
Married	4936 (92.33)	4284 (93.89)	120 (75.00)	532 (85.39)	
Rural, n(%)					.022
Urban	1910 (35.73)	1656 (36.29)	42 (26.25)	212 (34.03)	
Rural	3436 (64.27)	2907 (63.71)	118 (73.75)	411 (65.97)	
Drink, n(%)					.003
No	3438 (64.31)	2897 (63.49)	119 (74.38)	422 (67.74)	
Yes	1908 (35.69)	1666 (36.51)	41 (25.62)	201 (32.26)	
Smoke, n(%)					.360
No	3654 (68.35)	3136 (68.73)	106 (66.25)	412 (66.13)	
Yes	1692 (31.65)	1427 (31.27)	54 (33.75)	211 (33.87)	
Education, n(%)					<.001
Below primary school	2177 (40.74)	1700 (37.26)	115 (72.33)	362 (58.20)	
Primary school	1214 (22.72)	1056 (23.15)	29 (18.24)	129 (20.74)	
Middle school	1280 (23.96)	1175 (25.76)	11 (6.92)	94 (15.11)	
High school or above	672 (12.58)	631 (13.83)	4 (2.52)	37 (5.95)	
Chronic, n(%)					.031
No	2119 (39.64)	1842 (40.37)	57 (35.62)	220 (35.31)	
Yes	3227 (60.36)	2721 (59.63)	103 (64.38)	403 (64.69)	
Depression, n(%)					<.001
No	3864 (72.28)	3347 (73.35)	100 (62.50)	417 (66.93)	
Yes	1482 (27.72)	1216 (26.65)	60 (37.50)	206 (33.07)	
BMI, n(%)					<.001
18.5–23.9	2762 (51.66)	2383 (52.22)	80 (50.00)	299 (47.99)	
<18.5	255 (4.77)	51 (1.12)	58 (36.25)	146 (23.43)	
≥24	2329 (43.57)	2129 (46.66)	22 (13.75)	178 (28.57)	
Age, Mean ± SD	57.22 ± 8.68	55.79 ± 7.87	69.79 ± 7.20	64.52 ± 8.46	<.01
Sleep duration, Mean ± SD	6.72 ± 1.64	6.75 ± 1.61	6.47 ± 2.01	6.52 ± 1.78	<.01

### 3.2. Associations of sarcopenia and sleep duration with depressive symptoms

As shown in Figure 2, multivariable Cox proportional hazards models were employed to assess the relationships of sarcopenia status and sleep duration with the risk of developing depressive symptoms. Across three models with progressive adjustment for confounders, both sarcopenia and possible sarcopenia were significantly associated with an increased risk of depressive symptoms, while sleep duration showed a significant inverse association with this risk. Specifically, in Model 1 (unadjusted), the HR for sarcopenia was 1.57 (95% CI:1.21–2.02, *P* = .001), and for possible sarcopenia was 1.29 (95% CI:1.10–1.51, *P* = .002). The HR for sleep duration was 0.92 (95% CI:0.89–0.95, *P* < .001), indicating an 8% reduction in the risk of depressive symptoms per unit increase in sleep duration. In Model 2, adjusted for age, sex, marital status, educational level, and residence, the HR for sarcopenia was 1.38 (95% CI: 1.06–1.80, *P* = .018), for possible sarcopenia was 1.23 (95% CI:1.05–1.44, *P* = .012), and for sleep duration was 0.92 (95% CI: 0.89–0.95, *P* < .001). After further adjustment for chronic diseases, smoking, alcohol use, and BMI in Model 3, the association remained significant: the HR was 1.36 (95% CI: 1.04–1.78, *P* = .026) for sarcopenia, 1.21 (95% CI:1.02–1.44, *P* = .027) for possible sarcopenia, and 0.93 (95% CI:0.90–0.96, *P* < .001) for sleep duration. The results demonstrate that sarcopenia is significantly associated with an increased risk of incident depressive symptoms, and this association remains statistically significant after adjusting for varying degrees of confounding factors. Meanwhile, longer sleep duration is correlated with a lower risk of depressive symptoms.

**Figure 2.**
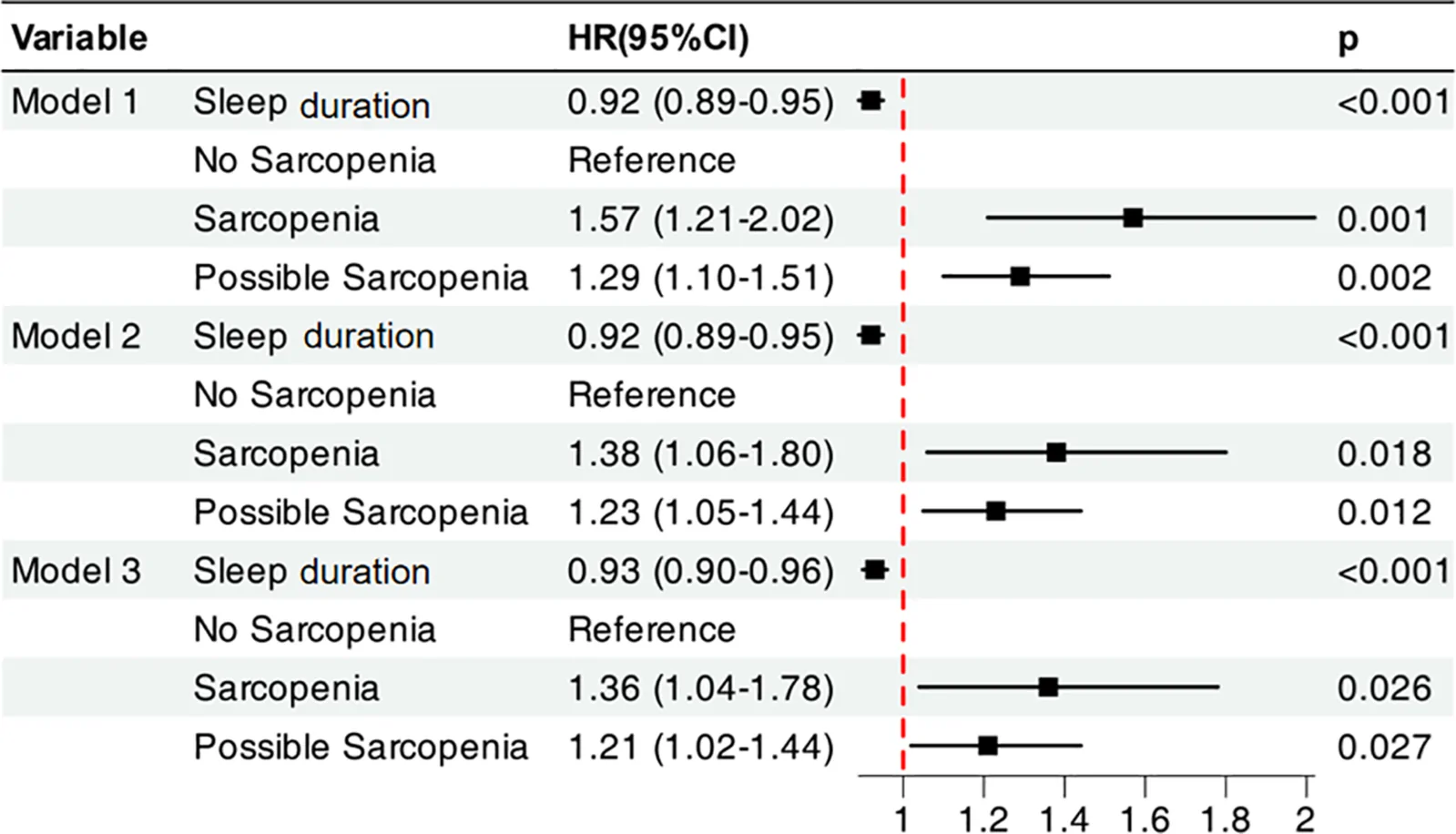
Hazard ratios (HRs) and 95% confidence intervals (CIs) for depression risk according to sleep duration and sarcopenia status in Cox regression models. CI = confidence interval, HR = hazard ratio.

### 3.3. Nonlinear associations between sarcopenia indicators and depressive symptoms

As shown in Figure 3, RCS analysis revealed no significant nonlinear associations between male ASMI, HGS, SPPB, and depressive symptoms (*P* for nonlinearity = .472, .870, .839, respectively). No gender differences were observed in these associations. Table A2, Supplemental Digital Content 2 presents the subgroup analysis results of the association between sarcopenia and depressive symptoms, indicating that no relevant factors significantly modified this relationship.

**Figure 3.**
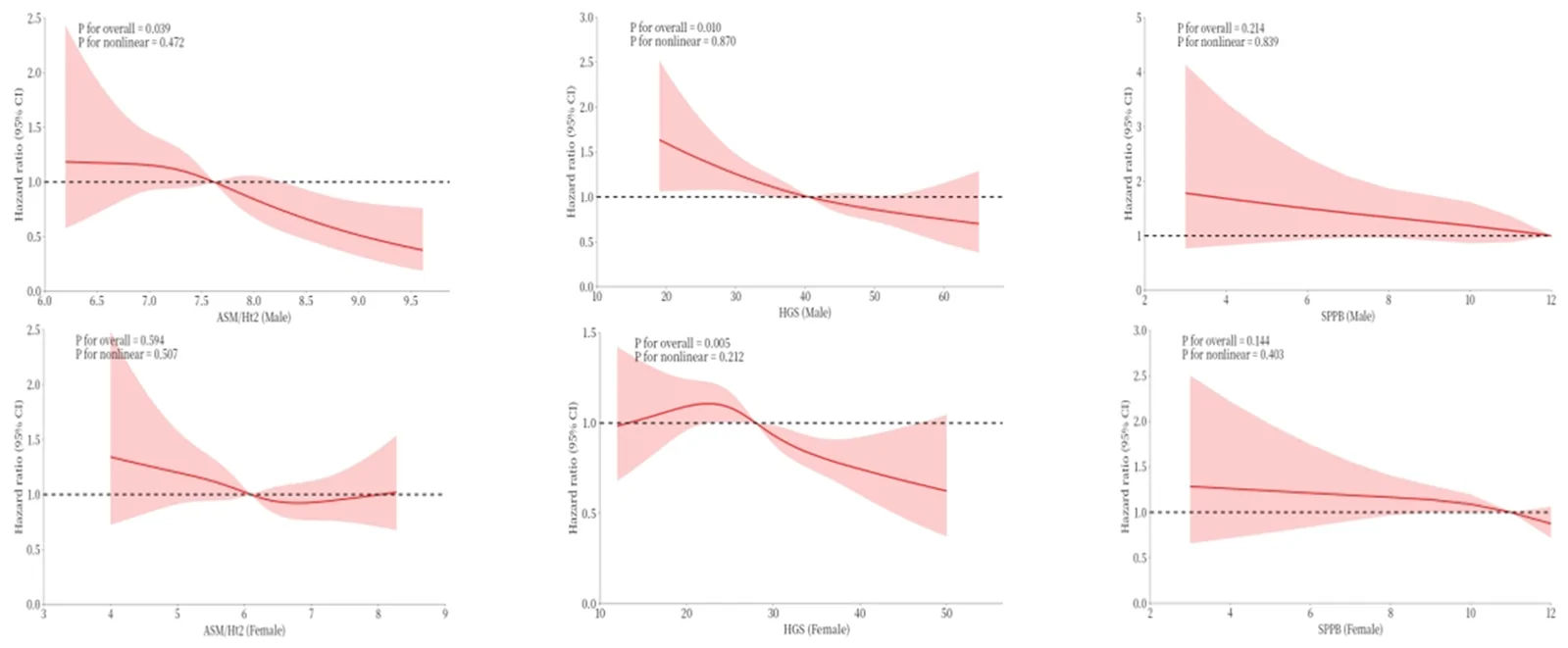
Restricted cubic spline (RCS) analysis of the association between sarcopenia indicators (ASMI, handgrip strength, SPPB) and depression risk. ASMI = appendicular skeletal muscle mass index, CI = confidence interval, HR = hazard ratio, RCS = restricted cubic spline, SPPB = short physical performance battery.

### 3.4. Sex-specific association between sleep duration and depressive symptoms

As shown in Figure 4, the RCS analysis stratified by gender revealed a statistically significant overall association between sleep duration and depressive symptoms (*P* for overall <.01). For the nonlinear relationship, a significant U-shaped association was observed in males (*P* for nonlinearity = .039), with approximately 7 hours representing the optimal sleep duration. However, no significant nonlinear association was detected in females (*P* for nonlinearity = .16). Table A3, Supplemental Digital Content 3 presents the subgroup analysis of the association between sleep duration and depressive symptoms, indicating that no relevant factors significantly modified this relationship.

**Figure 4.**
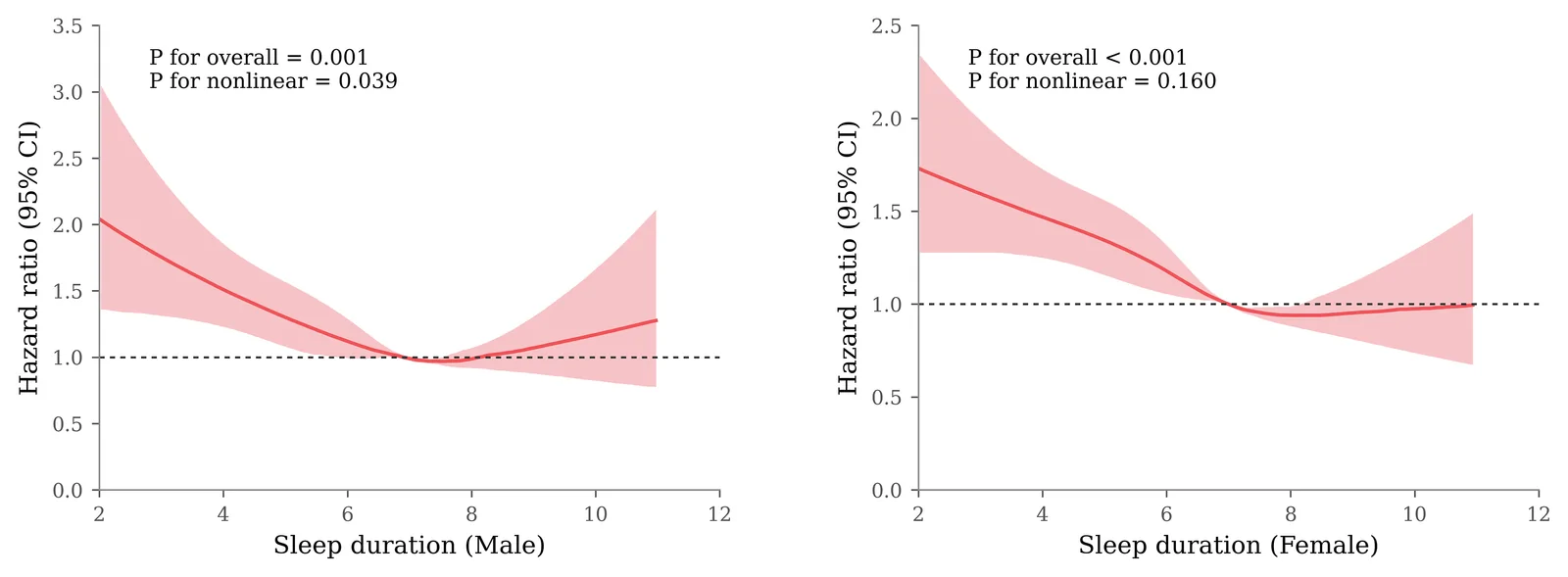
Sex-specific restricted cubic spline (RCS) analysis of the association between sleep duration and depression risk. CI = confidence interval, RCS = restricted cubic spline.

### 3.5. Mediating role of sleep duration in the Sarcopenia-depression pathway

As shown in Figure 5, mediation analysis was conducted to examine the potential mediating role of sleep duration in the association between sarcopenia and depressive symptoms. In the unadjusted model, the total effect of sarcopenia on depressive symptoms was significant (β = 0.26, *P* < .001), and the indirect effect through sleep duration was statistically significant but small (β = 0.019, *P* < .001). The path coefficients were as follows: path a (sarcopenia → sleep duration) β = −0.24, *P* < .001; path b (sleep duration → depressive symptoms) β = −0.08, *P* < .001; and path c’ (direct effect) β = 0.24, *P* < .001. After adjustment for covariates including gender, marital status, residence, alcohol consumption, smoking status, education level, chronic diseases, BMI, and age, the total effect remained significant (β = 0.19, *P* = .019). However, the mediating role of sleep duration was substantially attenuated. The adjusted path coefficients were as follows: path a became non-significant (β = −0.08, *P* = .283), while path b remained significant (β = −0.07, *P* < .001) and the direct effect of sarcopenia on depressive symptoms persisted (β = 0.18, *P* = .026). Importantly, after adjustment, the indirect effect through sleep duration was not statistically significant (*P* = .302). These results indicate that the association between sarcopenia and depressive symptoms remained after adjustment, but the indirect effect through sleep duration was not statistically significant.

**Figure 5.**
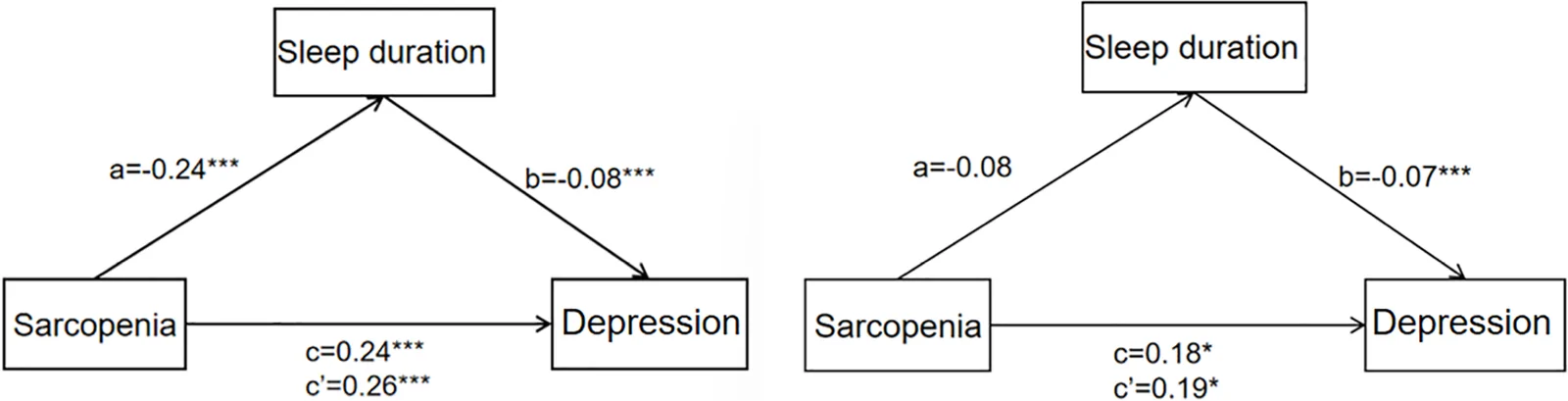
Mediation analysis of sleep duration in the association between sarcopenia and depression. Path coefficients are shown with 95% CIs. Model adjusted for gender, marital status, residence, alcohol consumption, smoking, education, chronic diseases, BMI, and age. BMI = Body mass index, CI = confidence interval.

### 3.6. Sensitivity analysis using continuous CESD-10 score

As shown in Table 2, to assess whether the main findings were sensitive to the dichotomization of depressive symptoms (CESD-10 ≥ 10), we repeated the analyses using the continuous CESD-10 score as the outcome in linear regression models. The results were consistent with the primary Cox regression analyses. In the fully adjusted model (Model 3), each one-hour increase in sleep duration was significantly associated with a decrease in CESD-10 score (β = −0.28, 95% CI: −0.33–−0.24, *P* < .001), and sarcopenia was significantly associated with an increase in CESD-10 score (β = 0.33, 95% CI:0.10 to 0.56, *P* = .005). These findings confirm that our conclusions are robust and not dependent on the specific CESD-10 cutoff threshold.

**Table 2:** Linear regression analysis of the associations of sleep duration and sarcopenia with continuous CESD-10 score.

	*β*	95%CI	*P*
Model 1			
Sleep duration	−0.30	−0.30 (−0.35–−0.26)	<.001
Sarcopenia	0.56	0.56 (0.35–0.77)	<.001
Model 2			
Sleep duration	−0.29	−0.29 (−0.34–−0.25)	<.001
Sarcopenia	0.37	0.37 (0.14–0.60)	.002
Model 3			
Sleep duration	−0.28	−0.28 (−0.33–−0.24)	<.001
Sarcopenia	0.33	0.33 (0.10–0.56)	.005

## 4. Discussion

This study utilized data from the CHARLS to systematically investigate the association between sarcopenia and the risk of depressive symptoms in middle-aged and older adults, as well as the potential mediating role of sleep duration. The findings demonstrated that both confirmed sarcopenia and its preliminary stage (possible sarcopenia) were significantly associated with an increased risk of depressive symptoms. These associations remained robust after sequential adjustment for demographic characteristics, lifestyle factors, and health status. Additionally, sleep duration was significantly correlated with depressive symptom risk. Mediation analysis indicated that although sleep duration exhibited a weak mediating effect in the unadjusted model, this effect became nonsignificant after controlling for confounders. These results suggest that the association between sarcopenia and depressive symptoms remained after adjustment, while the mediating role of sleep duration may be confounded or obscured by other factors.

Numerous cross-sectional studies have reported a significantly higher prevalence of depressive symptoms among individuals with sarcopenia compared to those without. For instance, a matched case-control study conducted in Thailand found that older adults with severe sarcopenia had significantly higher depression scores than nonsarcopenic controls, with the severity of depression being more pronounced in severe cases.^[17]^ A community-based study further revealed a significant positive correlation between sarcopenia and depressed mood, showing that the risk of depression increased with the severity of sarcopenia, with odds ratios ranging from 1.49 to 3.62.^[18]^ Data from the National Health and Nutrition Examination Survey in the United States also indicated a close association between sarcopenic obesity and depression.^[19]^ A meta-analysis by Li et al confirmed a significant positive correlation, reporting a pooled prevalence of depression of 28% among sarcopenic individuals and a 57% higher risk of depression after adjusting for confounders compared to nonsarcopenic individuals.^[1]^ Furthermore, research by Soysal et al suggested a positive correlation between the severity of sarcopenia and the risk of depression, where patients diagnosed with severe sarcopenia had a significantly higher risk than those only meeting the criteria for “possible sarcopenia.”^[20]^ These findings are largely consistent with the results of the present study. Research in specific populations further elucidates the complex relationship. Studies on stroke rehabilitation patients indicated that sarcopenia, particularly decreased HGS, was significantly associated with the improvement of depressive symptoms.^[21]^ In diabetic patients, depressive symptoms were found to indirectly increase the risk of sarcopenia through factors like malnutrition.^[22]^ Several studies suggest that the association between sarcopenia and depression might be more pronounced in women. For example, a Korean study reported a higher risk of depression among women with sarcopenia compared to men.^[18]^ Another study also indicated a significantly higher prevalence of depressive symptoms in female sarcopenic patients than in males.^[23]^ However, our study did not find a statistically significant gender difference in the association between sarcopenia and depressive symptoms. The inclusion of a broader age range in our study, compared to the aforementioned studies focusing primarily on older elderly populations, is a key methodological difference that may explain this discrepancy.

The association between sarcopenia and depression is underpinned by complex and potentially interrelated biological mechanisms. From a neurophysiological perspective, sarcopenia may contribute to the development of depression and cognitive decline, which in turn can lead to frailty. Mediation analyses suggest that both depression and cognitive function act as partial mediators in the pathway from sarcopenia to frailty, with depression exerting a significant effect across all sarcopenia subgroups.^[12]^ Regarding pathophysiological mechanisms, sarcopenia is often accompanied by chronic systemic inflammation and metabolic dysregulation. These systemic alterations may be associated with changes in the central nervous system, thereby increasing the risk of depression. For instance, the accumulation of circulating advanced glycation end products, associated with declining muscle function, may promote depression through pro-inflammatory pathways.^[24,25]^ From a biometabolic standpoint, a reduced basal metabolic rate (BMR) is closely linked to an increased risk of depression. Sarcopenia may indirectly promote depression by lowering BMR; research indicates that each unit increase in BMR is associated with an 11% reduction in depression risk, suggesting that the decline in metabolic levels consequent to sarcopenia could be a significant mechanism.^[26]^ Furthermore, as indicated by Jo et al sarcopenia may induce cognitive decline through an imbalance in myokine secretion resulting from reduced skeletal muscle mass, providing another potential pathway for the development of depression.^[27]^ It is important to note that a bidirectional relationship likely exists between sarcopenia and depression. When sarcopenia is the exposure, the risk of incident depression significantly increases. A longitudinal study demonstrated that individuals with sarcopenia had a markedly higher risk of developing depression later on.^[28]^ Conversely, when depression is the exposure, the risk of sarcopenia is also elevated. A meta-analysis showed that depressive symptoms increase the risk of sarcopenia, an association that appears more pronounced in individuals with chronic diseases.^[29]^ This bidirectional interplay is further highlighted by research showing that sarcopenia is associated with poorer cardiovascular health partly through the mediating role of depression, underscoring the importance of the sarcopenia-depression pathway in overall health.^[30]^ This network of complex interactions suggests that sarcopenia is associated with health status not only directly through physiological decline but also indirectly through psychological and cognitive pathways.

Multiple studies consistently indicate a significant association between sleep patterns and the risk of depression, demonstrating a clear dose-response trend. Compared to middle-aged and older adults with healthy sleep patterns, those with intermediate sleep patterns have a 2.28-fold increased risk of depression, while the risk escalates to 5.60-fold among those with poor sleep patterns.^[31]^ This risk is associated not only with overall sleep patterns but also specifically with sleep duration. A dose-response meta-analysis indicated that both excessively short and long nighttime sleep durations are associated with an increased incidence of depression, suggesting a U-shaped relationship between sleep duration and depression risk,^[11]^ a finding that aligns with the significant U-shaped association observed particularly in males within our study. The link between sleep disturbances and depression may operate through various physiological and psychological pathways. From a physiological perspective, sleep disruption, such as increased wake time after sleep onset, may activate inflammatory response pathways. Research has found that sleep disturbance leads to elevated levels of inflammatory markers like interferon-gamma, which is directly associated with depressive symptoms and appears more pronounced in women.^[32,33]^ Furthermore, a U-shaped association between sleep duration and mortality exists among individuals with preexisting depression, suggesting that sleep dysregulation may be associated with worse overall mental health through mechanisms such as autonomic nervous system dysfunction.^[34]^ Regarding psychological and cognitive mechanisms, sleep quality appears to be more critical for emotional regulation than absolute sleep duration. Sleep fragmentation is linked to cognitive deterioration and can exacerbate depressive symptoms through cycles of negative thinking, including repetitive worry and cognitive biases.^[35,36]^ This highlights the complex interplay between sleep physiology, cognitive-emotional processes, and mental health outcomes.

A complex bidirectional association exists between sleep and depression. One study found that actigraphy-estimated 24-hour activity rhythms, sleep timing, and self-reported sleep quality all demonstrated bidirectional predictive relationships with depressive symptoms.^[37]^ This bidirectional link is further supported by longitudinal evidence; individuals with depression have a 72% increased risk of developing sleep disturbances, while the risk of worsening depressive symptoms is also elevated by 73%.^[10]^ The association between sleep and depression may differ by gender, with some research suggesting a stronger effect of insomnia on depression in men.^[38]^ From a life-course perspective, both the duration and timing of sleep problems are crucial. The Singapore Chinese Health Study revealed that short sleep duration in midlife, regardless of whether it persisted into late life, was associated with an increased risk of late-life depression. Conversely, concurrently short or long sleep duration in late life was associated with co-existing depressive symptoms.^[39]^ Intervention studies suggest that improving sleep quality could be an effective strategy for reducing depression risk. A 10-year cohort study indicated that improving sleep quality significantly lowered the risk of depression among those with intermediate baseline sleep quality, potentially through indirect pathways such as alleviating pain and enhancing physical function.^[40]^

Sleep has been postulated to play a potential mediating role in the association between sarcopenia and depressive symptoms, a hypothesis supported by indirect evidence and plausible pathways in the existing literature. For instance, individuals with sarcopenia often exhibit abnormal sleep duration, such as prolonged sleep,^[41]^ which is itself independently associated with an elevated risk of depression.^[42]^ Furthermore, the chronic inflammatory state characteristic of sarcopenia, marked by elevated levels of pro-inflammatory cytokines like Interleukin-6 and Interleukin-1β, is thought to potentially contribute to depression partly through the disruption of sleep, possibly mediated by mechanisms such as gut microbiota dysbiosis.^[43,44]^ Within a broader network of interrelated factors, sarcopenia, sleep disturbances, and depression are considered to collectively influence frailty through chain reactions. However, the findings from our study present a more nuanced picture. In the unadjusted model, sleep duration demonstrated only a weak mediating effect. Crucially, after adjusting for key covariates including age, comorbidities, and lifestyle factors, the mediating effect of sleep duration was no longer statistically significant. This indicates that while theoretical and observed associations link sleep duration with both conditions, its role as an independent mediator may not be robust when accounting for potential confounders.

The bidirectional relationship between sleep and depression warrants particular attention in interpreting our mediation findings. Although we excluded participants with baseline depressive symptoms to establish temporal precedence, subclinical depressive symptoms at baseline below the diagnostic threshold may have influenced both sleep duration and subsequent depression risk. Conversely, sleep disturbances may represent an early prodromal manifestation of depression rather than a true mediator. Previous meta-analyses have demonstrated bidirectional associations between sleep disturbances and depression in older adults, with depression predicting future sleep problems and vice versa. Our mediation results should therefore be interpreted with caution, as the single baseline measurement of sleep duration cannot fully capture this dynamic and potentially reciprocal interplay. Future studies with repeated assessments of both sleep and depressive symptoms are needed to disentangle the temporal ordering of this relationship.

This study has several limitations. First, despite the prospective design and adjustment for multiple confounders, the observational nature cannot fully rule out residual confounding or reverse causality. Although we excluded participants with baseline depressive symptoms, subclinical depression may have influenced both sleep and subsequent depression risk. The well-documented bidirectional relationship between sleep and depression further complicates causal interpretation of the mediation findings. Second, sarcopenia was assessed using a validated anthropometric equation rather than dual-energy X-ray absorptiometry, which may introduce measurement error and attenuation bias. Sleep duration was self-reported and limited to duration only; other dimensions (e.g., sleep quality, insomnia symptoms) were unavailable and may partly explain the weak mediating effect. Third, important confounders including physical activity, diet, antidepressant use, pain, and cognitive function were not available in the CHARLS dataset, and their absence may have led to overestimation of the observed associations. Fourth, the exclusion of participants with missing data and baseline depression substantially reduced the sample (from 17,708–5346). While we used IPW to address selection bias, we did not perform multiple imputation due to nonrandom missingness, which may limit generalizability. Fifth, both exposure and mediator were measured only at baseline, precluding definitive causal inference regarding the mediation pathway; this analysis should therefore be considered exploratory. Finally, as the findings are derived from a Chinese population, generalizability to other populations requires further validation.

## 5. Conclusion

In the middle-aged and older Chinese population, sarcopenia was independently associated with an increased risk of incident depressive symptoms. Sleep duration did not significantly mediate this association after adjustment. These exploratory findings should be interpreted cautiously. Although sleep duration was independently associated with depressive symptom risk, its indirect association between sarcopenia and depressive symptoms was not statistically significant after adjustment and may be susceptible to confounding. Therefore, interventions targeting sarcopenia prevention and management, along with sleep health promotion, may have beneficial effects on mental well-being in this population.

## Acknowledgments

We would like to thank all the CHARLS staff for their valuable contributions to data collection and management. This research received no specific grant from any funding agency in the public, commercial, or not-for-profit sectors. The authors declare that they have no competing interests.

## Author contributions

**Conceptualization:** Jiaran Jiang, Quan Zhou.

**Data curation:** Jiaran Jiang.

**Formal analysis:** Jiaran Jiang.

**Investigation:** Jiaran Jiang, Chuanxia Zhang.

**Methodology:** Jiaran Jiang, Chuanxia Zhang.

**Software:** Jiaran Jiang, Quan Zhou.

**Validation:** Jiaran Jiang, Yiming Ma.

**Visualization:** Jiaran Jiang.

**Funding acquisition:** Quan Zhou.

**Project administration:** Quan Zhou.

**Resources:** Quan Zhou.

**Supervision:** Quan Zhou.

**Writing – original draft:** Jiaran Jiang.

**Writing – review & editing:** Quan Zhou, Chuanxia Zhang, Yiming Ma.






