# Supplementary material for: Sleep duration, sarcopenia, and incident depressive symptoms in Chinese middle-aged and older adults: A prospective cohort study based on CHARLS
Source: Medicine (Baltimore). 2026 Oct 23;105(43):e51177. doi: 10.1097/MD.0000000000051177 (PMC13706259; doi:10.1097/MD.0000000000051177)
Supplement: Supplementary file 1 [file medi-105-e51177-s001.docx]

****Table A1. Variance Inflation Factors (VIF) for predictor variables in the multivariate model.****

| Variable | VIF | 1/VIF |
| --- | --- | --- |
| Gender | 1.99 | 0.502 |
| Education 2 | 1.77 | 0.566 |
| Education 3 | 1.62 | 0.618 |
| smoke | 1.55 | 0.647 |
| Education 4 | 1.42 | 0.706 |
| Drink | 1.37 | 0.731 |
| Age | 1.36 | 0.735 |
| Sarcopenia | 1.17 | 0.858 |
| Marry | 1.08 | 0.924 |
| BMI | 1.07 | 0.937 |
| Rural | 1.07 | 0.937 |
| Chronic | 1.03 | 0.968 |

Note: Mean VIF = 1.37.
