# Supplementary material for: Sleep duration, sarcopenia, and incident depressive symptoms in Chinese middle-aged and older adults: A prospective cohort study based on CHARLS
Source: Medicine (Baltimore). 2026 Oct 23;105(43):e51177. doi: 10.1097/MD.0000000000051177 (PMC13706259; doi:10.1097/MD.0000000000051177)
Supplement: Supplementary file 2 [file medi-105-e51177-s002.docx]

****Table A2. Subgroup analysis of the association between sarcopenia and depression.****

| Variables | n (%) | Non sarcopenia | Sarcopenia | HR (95%CI) | P | P for interaction |
| --- | --- | --- | --- | --- | --- | --- |
|  |  |  |  |  |  |  |
| All patients | 5346 (100.00) | 1216/4563 | 266/783 | 1.23 (1.04 ~ 1.44) | 0.013 |  |
| Gender |  |  |  |  |  | 0.671 |
| Male | 2696 (50.43) | 469/2305 | 108/391 | 1.35 (1.04 ~ 1.76) | 0.027 |  |
| Female | 2650 (49.57) | 747/2258 | 158/392 | 1.18 (0.96 ~ 1.45) | 0.109 |  |
| Marry |  |  |  |  |  | 0.267 |
| Others | 410 (7.67) | 92/279 | 46/131 | 1.16 (0.77 ~ 1.76) | 0.472 |  |
| Married | 4936 (92.33) | 1124/4284 | 220/652 | 1.23 (1.03 ~ 1.47) | 0.022 |  |
| Rural |  |  |  |  |  | 0.999 |
| Urban | 1910 (35.73) | 340/1656 | 68/254 | 1.30 (0.95 ~ 1.77) | 0.098 |  |
| Rural | 3436 (64.27) | 876/2907 | 198/529 | 1.19 (0.98 ~ 1.44) | 0.078 |  |
| Drink |  |  |  |  |  | 0.471 |
| No | 3438 (64.31) | 862/2897 | 194/541 | 1.20 (0.99 ~ 1.44) | 0.061 |  |
| Yes | 1908 (35.69) | 354/1666 | 72/242 | 1.35 (0.97 ~ 1.88) | 0.076 |  |
| Smoke |  |  |  |  |  | 0.467 |
| No | 3654 (68.35) | 890/3136 | 180/518 | 1.18 (0.98 ~ 1.43) | 0.087 |  |
| Yes | 1692 (31.65) | 326/1427 | 86/265 | 1.38 (1.01 ~ 1.88) | 0.042 |  |
| Education |  |  |  |  |  | 0.416 |
| Below primary school | 2177 (40.74) | 591/1700 | 181/477 | 1.14 (0.93 ~ 1.40) | 0.196 |  |
| Primary school | 1214 (22.72) | 248/1056 | 55/158 | 1.53 (1.07 ~ 2.19) | 0.018 |  |
| Middle school | 1280 (23.96) | 265/1175 | 25/105 | 1.30 (0.79 ~ 2.13) | 0.302 |  |
| High school or above | 672 (12.58) | 112/631 | 5/41 | 0.98 (0.35 ~ 2.78) | 0.973 |  |
| Chronic |  |  |  |  |  | 0.964 |
| No | 2119 (39.64) | 391/1842 | 73/277 | 1.19 (0.86 ~ 1.64) | 0.284 |  |
| Yes | 3227 (60.36) | 825/2721 | 193/506 | 1.23 (1.02 ~ 1.49) | 0.029 |  |
| BMI |  |  |  |  |  | 0.480 |
| 18.5-23.9 | 2762 (51.66) | 627/2383 | 134/379 | 1.36 (1.10 ~ 1.68) | 0.005 |  |
| <18.5 | 255 (4.77) | 19/51 | 66/204 | 0.88 (0.46 ~ 1.66) | 0.691 |  |
| ≥ 24 | 2329 (43.57) | 570/2129 | 66/200 | 1.13 (0.86 ~ 1.50) | 0.380 |  |
| Age |  |  |  |  |  | 0.190 |
| 45-60 | 3180 (61.25) | 837/3037 | 46/143 | 0.97 (0.69 ~ 1.36) | 0.876 |  |
| 61-75 | 1831 (35.27) | 315/1312 | 183/519 | 1.36 (1.11 ~ 1.66) | 0.002 |  |
| >75 | 181 (3.49) | 17/67 | 35/114 | 0.99 (0.52 ~ 1.89) | 0.974 |  |
| HR: Hazard Ratio, CI: Confidence Interval | | | | | | |
