# Supplementary material for: Sleep duration, sarcopenia, and incident depressive symptoms in Chinese middle-aged and older adults: A prospective cohort study based on CHARLS
Source: Medicine (Baltimore). 2026 Oct 23;105(43):e51177. doi: 10.1097/MD.0000000000051177 (PMC13706259; doi:10.1097/MD.0000000000051177)
Supplement: Supplementary file 3 [file medi-105-e51177-s003.docx]

****Table A3. Subgroup analysis of the association between sleep duration and depression.****

| Variables | n (%) | HR (95%CI) | P | P for interaction |
| --- | --- | --- | --- | --- |
|  |  |  |  |  |
| All patients | 5312 (100.00) | 0.92 (0.90 ~ 0.95) | <.001 |  |
| Gender |  |  |  | 0.560 |
| Male | 2696 (50.43) | 0.93 (0.89 ~ 0.98) | 0.006 |  |
| Female | 2650 (49.57) | 0.92 (0.88 ~ 0.95) | <.001 |  |
| Marry |  |  |  | 0.804 |
| Others | 410 (7.67) | 0.91 (0.84 ~ 0.99) | 0.022 |  |
| Married | 4936 (92.33) | 0.93 (0.90 ~ 0.96) | <.001 |  |
| Rural |  |  |  | 0.921 |
| Urban | 1910 (35.73) | 0.92 (0.87 ~ 0.98) | 0.009 |  |
| Rural | 3436 (64.27) | 0.93 (0.89 ~ 0.96) | <.001 |  |
| Drink |  |  |  | 0.476 |
| No | 3438 (64.31) | 0.93 (0.90 ~ 0.96) | <.001 |  |
| Yes | 1908 (35.69) | 0.91 (0.86 ~ 0.96) | 0.001 |  |
| Smoke |  |  |  | 0.724 |
| No | 3654 (68.35) | 0.92 (0.89 ~ 0.96) | <.001 |  |
| Yes | 1692 (31.65) | 0.93 (0.88 ~ 0.99) | 0.014 |  |
| Education |  |  |  | 0.393 |
| Below primary school | 2177 (40.74) | 0.94 (0.91 ~ 0.98) | 0.003 |  |
| Primary school | 1214 (22.72) | 0.90 (0.84 ~ 0.96) | 0.002 |  |
| Middle school | 1280 (23.96) | 0.88 (0.81 ~ 0.95) | 0.001 |  |
| High school or above | 672 (12.58) | 0.95 (0.82 ~ 1.09) | 0.466 |  |
| Chronic |  |  |  | 0.732 |
| No | 2119 (39.64) | 0.92 (0.87 ~ 0.97) | 0.002 |  |
| Yes | 3227 (60.36) | 0.93 (0.90 ~ 0.96) | <.001 |  |
| BMI |  |  |  | 0.530 |
| 18.5-23.9 | 2762 (51.66) | 0.93 (0.89 ~ 0.97) | <.001 |  |
| <18.5 | 255 (4.77) | 0.97 (0.87 ~ 1.09) | 0.641 |  |
| ≥ 24 | 2329 (43.57) | 0.91 (0.87 ~ 0.96) | <.001 |  |
| Age |  |  |  | 0.353 |
| 45-60 | 3180 (61.25) | 0.93 (0.89 ~ 0.97) | <.001 |  |
| 61-75 | 1831 (35.27) | 0.91 (0.87 ~ 0.95) | <.001 |  |
| >75 | 181 (3.49) | 1.01 (0.88 ~ 1.16) | 0.920 |  |
| HR: Hazard Ratio, CI: Confidence Interval | | | | |
